# Dynamics of economic growth: Uncertainty treatment using differential inclusions

**DOI:** 10.1016/j.mex.2019.02.029

**Published:** 2019-02-28

**Authors:** Stanislaw Raczynski

**Affiliations:** Universidad Panamericana, Facultad de Ingeniería, Augusto Rodin 498, Ciudad de México, 03920, Mexico

**Keywords:** Differential inclusion solver, Reachable set determination, Mathematical model, Economic model, System dynamics, Risk analysis, Economic growth, Simulation, Differential inclusions, Uncertainty

## Abstract

The article is focused on applications of the differential inclusions to the models of economic growth, rather than the model building. The models are taken from the known literature, and some modifications are introduced to reflect an additional inertia. The aim is to treat the uncertainty in the model parameters by using differential inclusions instead of the stochastic approach. The reachable sets for the models are shown, to assess the possible ranges of the outcome with given parameters uncertainty. The approach may be interpreted as a generalization to the system dynamics methodology, providing attainable sets instead of single model trajectory and simple sensitivity analysis. A comparison with Powersim risk analysis is provided. The models of Solow and Swan, Mankiw, Bhattacharya, Romer and Weil are used. A brief review of the models is given, and several examples of simple simulations, differential inclusion applications and optimization are presented.

## Graphical abstract

**Figure:**
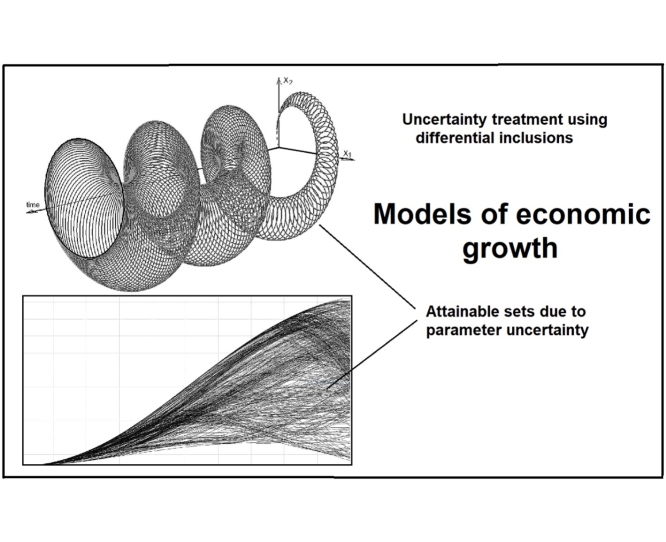

**Specifications Table**

**Table:** 

**Subject Area:**	**Engineering, mathematics, computer science**
**More specific subject area:**	*Differential inclusions applied to model of economic growth*
**Method name:**	*Differential inclusion solver, reachable set determination, mathematical model*
**Name and reference of original method:**	Differential inclusions, uncertainty in models of economic growth Aubin J.P., Chen L., Dordan O. (2014) Tychastic Measure of Viability Risk. Springer International Publishing, Switzerland, DOI: doi.org/10.1007/978-3-319-08129-8, ISBN: 978-3-319-08128-1. Aubin J.P., Cellina A. (1984) Differential Inclusions. Springer Verlag, Berlin, DOI: 10.1007/978-3-642-69512-4, ISBN: 978-3-642-69514-8. Basu D. (2009) Economic Models: Methods, Theory and Applications. World Scientific. Berkovitz L.D. (1964) Variational approach to differential games. Princeton Univ. Press, Princeton NJ. Bernanke B.S., Gürkaynak R.S. (2002) Is Growth Exogenous? Taking Mankiw, Romerand Weil Seriously. 16, MIT Press, URL: http://www.nber.org/books/bern02-1, ISBN: 0-262-02520-5. Bhattacharya P. C. (2007) Informal Sector, Income Inequality and Economic Development. Centre for Economic Reform and Transformation. Bhattacharya P. C. (1998) Migration, employment and development: a three-sector analysis. Journal of International Development, 10(7):899-921. Bhattacharya P. C. (1994) A multi-sector model of LDC. Scottish Journal of Political Economy, 41(3):225-255. Bhattacharya P. C. (1993) Rural-urban migration in economic development. Journal of Economic Surveys, 7(3):243-281. Byrknes A.H. (1996) Powersim tutorial 1: Introductory course. Powersim Corporation, ISBN: 1889353000. Cobb C.W., Douglas P.H. (1928) A Theory of Production. American Economic Review, 18 (Supplement):139-165. Diamand R.W., Spencer B.J. (2008) Trevor Swan And The Neoclassical Growth Model. Report NBER Papers in Economic Fluctuations and Growth. Fudenberg D., Tirole J. (1991) Game Theory. MIT Press, Cambridge MA. Gini C. (1912) Variabilità e mutabilità. (Pizetti, E.; Salvemini, T., eds., eds.), Libreria Eredi Virgilio Veschi, Rome. Grigorieva S.V., Ushakov V.N. (2000) Use Finite Family of Multivalued Maps for Constructing Stable Absorption Operator. Topological Methods in Nonlinear Analysis, Juliusz Schauder Center, 50(1). Guttorp P., Minin V.N. (1995) Stochastic Modeling of Scientific Data. Springer Science Business Media, ISBN/ISSN 10: 0412992817 Isaacs R. (1999) Differential Games. Dover Publications, Inc., New York, ISBN: 9780486406824. Kaldor N. (1957) A model of economic growth. Economic Journal, 67(268):591-624. Kaya V.P. (2004) Rostow's Stages of Development. Internet communication, URL: http://www.nvcc.edu/home/nvfordc/econdev/introduction/stages.html. Keynes M. (1964) The General Theory of Employment, Interest and Money. Harcourt Brace and Company, New York. Krbec P. (1986) On Nonparasite Solutions. Internet communication Equadiff 6. Brno J. E. Purkyne University, Department of Mathematics: 133-139, URL: http://eudml.org/doc/220003. Mankiw N.G. (1998) Principles of economic growth. Dryden Press, Fort Worth TX, ISBN: 0-03098238-3. Marchaud A. (1934) Sur les champs de demi-cones et les équations differielles du premier ordre. Bulletin de la Société mathématique de France, Société mathématique de France, 62. Petrosjan L., Zenkiewicz N.A. (1996) Game Theory. World Scientific Publishing Co., Inc. Plis A. (1961) Remark on measurable set-valued functions. Bulletin de l'Academie Polonaise des Science - Serie des Sciences Mathematiques, Astronomiques et Physiques, 9(12). Pontryagin L.S. (1962) The mathematical theory of optimal processes. Wiley Interscience, New York. Quincamoix M., Saint-Pierre P. (1995) An Algorithm for Viabilit y Kernels in Holderian Case: Appro ximation Approximation by Discrete Dynamical Systems. Discrete Dynamical Systems Journal of Mathematical Systems, Estimation, and Control, 5(1). Raczynski S. (2002) Differential Inclusion Solver. Conference paper: International Conference on Grand Challenges for Modeling and Simulation, The Society for Modeling and Simulation, San Antonio, TX. Ricardo D. (1996) Principles of Political Economy and Taxation. Amherst, NY. Saint-Pierre P. (1994) Approximation of the viability kernel. Applied Mathematics and Optimization, 29(2):187-209. Sato R. (1964) Harrod Domar Growth model. The Economic Journal, Wiley, Royal Economic Society, 74(294):380-387, URL: http://www.jstor.org/stable/2228485, DOI: 10.2307/2228485. Sentis R. (1978) Equations diferentielles a second membre mesurable. Bollettino dell'Unione Matematica Italiana, 15(B):724-742, ISBN/ISSN 1972-6724 Smith A. (1998) An Inquiry into the Nature and Causes of the Wealth of Nations. (Campbell R.H. and Skinnes A.S., eds.) Glasgow. Solan E., Vielle N. (2001) Quitting Games. Mathematics of Operations Research, INFORMS, 26(2):265-285. Solow R.M. (1956) 'A Contribution to the Theory of Economic Growth. Economics, MIT Press, 70(1):65-94, URL: http://www.jstor.org/stable/1884513. Swan T.W. (1956) Economic Growth and Capital Accumulation. Economic Record, 32(63):334-361. Turowicz A. (1963) Sur les zones d'emision des trajectoires et des quasitrajectoires des systemes de commande nonlineaires. Bulletin de l'Academie Polonaise des Science - Serie des Sciences Mathematiques, Astronomiques et Physiques, 11(2). Turowicz A. (1962) Sur les trajectoires et quasitrajectoires des systemes de commande nonlineaires. Bulletin de l'Academie Polonaise des Science - Serie des Sciences Mathematiques, Astronomiques et Physiques, 10(10). Walde K. (1999) A Model of Creative Destruction with Undiversifiable Risk and Optimising Households. The Economic Journal, 109(454):156-171, DOI: doi.org/10.1111/1468-0297.00423. Wazewski T. (1963) On an optimal control problem Differential Equations and Their Applications. Conference paper: Proceedings of the Conference held in Prague, Publishing House of the Czechoslovak Academy of Sciences, Prague. Wazewski T. (1962) Sur les systemes de commande non lineaires dont le contredomaine de commande n'est pas forcement convexe. Bulletin de l'Academie Polonaise des Science - Serie des Sciences Mathematiques, Astronomiques et Physiques, 10(1). Wazewski T. (1962) Sur une genralisation de la notion des solutions d'une equation au contingent. Bulletin de l'Academie Polonaise des Science - Serie des Sciences Mathematiques, Astronomiques et Physiques, 10(1). Wazewski T. (1961) Sur une condition equivalente a l'equation au contingent. Bulletin de l'Academie Polonaise des Science - Serie des Sciences Mathematiques, Astronomiques et Physiques, 9(12). Zaremba S.K. (1936) Sur les équations au paratingent. Bulletin des Sciences Mathematiques, 60.
**Resource availability:**	http://www.raczynski.com/solver/disolver.html

## Method details

The main topic of this article is the way the uncertainty in some economic growth models can be treated, and not the model building.

System dynamics (SD) approach to modeling and simulation of wide class of systems has been used along several decades, providing relevant insight on model properties and dynamic behavior [45]. Roughly speaking, the methodology consists in the user-friendly graphical model representation, which is used to generate ordinary differential equations (ODE) model, and to solve the equations to display the model evolution in time. Here, we discuss the influence of parameter uncertainty on the model dynamics. Such problems are frequently treated by stochastic modeling, where the uncertain parameters are random variables, see Guttorp and Minin [1]. Such probabilistic approach to uncertainty may provide useful results, including the information about the state variables deviations and distributions due to the uncertainty. However, observe that uncertainty is not the same as randomness. To make a stochastic analysis, we should know the probabilistic properties of the uncertain variables. Sometimes it is difficult or impossible to obtain such information. Moreover, the model parameters may not be random, but just uncertain, and may have no probability distribution at all. For example, the uncertain information about the demand in the stock market model may be the result of intentionally introduced false information. This uncertainty treatment is similar to the *tychastic control* definition, see Aubin et al. [2].

The contribution of this paper to the economic literature is to discuss how the mathematical concepts of differential inclusions and reachable sets can be applied in macroeconomic dynamics to handle certain kinds of uncertainty. We do not define any particular kind of uncertainty. Here, the only requirement is that the restrictions of uncertain parameters are known. This variables can represent, for example, the unexpected parameter changes due to the influence of external events, the intentionally generated false information about the economic parameters or a random, limited fluctuations. If the parameter could be treated as random, we do not need any corresponding probabilistic properties. In other words, the uncertain parameters may be random or not, as in the case of the tychastic variables.

In the method proposed here, we only need the information about the limits where the possible values of uncertain parameters belong. We do not require the uncertain parameters to be constant, treating them as functions of time. A brief comparison between the results obtained from the Powersim risk analysis and the attainable sets provided by the differential inclusion solver is given in sections 2 and 3.

As for the pros and cons of using differential inclusions (DI), note that DI approach is completely different from the most popular methods, based mainly on the stochastic approach, worse- or extreme-case studies. Thus, it is rather difficult to compare such different methods and results. Note that the DI approach discussed here is deterministic and provides the reachable sets due to the uncertainty and not any probabilistic properties of model trajectoris.

## Modeling tool: differential inclusions

In this section you can find some general remarks on differential inclusions. More detailed assumptions and exhaustive survey can be found in Aubin and Cellina [3]. The differential inclusion (DI) is defined by the following statement.

(1) $$dx/dt\in F(t,x(t)),\;\;x(0)\in X_{0}$$

where *F* is a mapping from *R*x*R^n^* to subsets of *R^n^* and *X_0_* is the initial set, also named *the set of admissible directions*. *R^n^* is the real n-dimensional Euclidean space, *x*∈*R^n^*, *t* is a real variable representing the model time. In the following *R* is equal to *R^1^*. Differential inclusion can be treated as a generalization of a differential equation. The difference is that in a differential equation the right hand *F* of the inclusion reduces to a point-valued function, and the inclusion becomes an equation sign.

Recall some historical facts. The early works on the DIs in the finite-dimensional case were published by Marchaud [4] and Zaremba [5]. The terminology used in these papers was slightly different from more recent works. Zaremba used the term *paratingent equation* and Marchaud called the same a *contingent equation*. Both of them did not suspect the importance of these concepts for future applications in the Control Theory. Moreover, the research was criticized by contemporary mathematicians and physicists as an abstract and useless work. In fact, Zaremba and Marchaud had already described the main properties of trajectories and reachable sets of control systems with convex sets of admissible directions, many years before those problems have been treated by the Control Theory. The later works of Turowicz and Wazewski provided more important results. Wazewski [[6], [7], [8]], Turowicz [9,10] and Plis [11] gave the generalization of the basic results to the case of non-convex sets of admissible directions (the set *F* in (1)). This is important in control systems analysis, in particular when the "bang-bang" type of control is used. In the terminology used by Wazewski the right-hand side of the differential inclusion is called **orientor field** and (1) is called *orientor condition*.

One of the most important concepts, widely discussed by Wazewski, is the *quasitrajectory* of an orientor field (weak solution to the DI). This concept is closely related to the "bang-bang" control and sliding regimes known in the automatic control theory. Wazewski [7] discussed the DIs derived from a control system, where the control variable belongs to a given set *C.* In this case, the set *F* of the right-hand side of the corresponding DI can be derived by parametrization, *F* being a mapping of *C.* In the article of Wazewski, both *C* and *F* need not be convex. This relation to control system will be discussed with more detail further on. The concept of the quasitrajectory, in the case of non-convex right-hand side of (1) has been commented later [12,13]. See also the book on differential inclusions of Aubin and Cellina [3].

A lot of relevant research on DIs in game theory has been done. Many of the works in the field use the Hamilton-Jacobi-Bellman's equations and the methods of the control theory, closely related to the DIs Grigorieva and Ushakov [14] consider the differential game of pursuit-evasion over a fixed time interval. The attainable set is appointed with the help of the stable absorption operator. A more general, variational approach to differential games can be found in Berkovitz [15]. The DIs are used by Solan and Vielle [16] to study the equilibrium payoffs in quitting games. For general problems of the Game Theory, consult, for example, Petrosjan and Zenkiewicz [17], Isaacs [18] or Fudenberg and Tirole [19].

To see a more rigorous mathematical theory we recommend the previously mentioned book of Aubin and Cellina [3].

A function *x(t)* that satisfies (1) almost everywhere over a given time interval, is called a *trajectory* of the DI. Sometimes such function is called a *solution* to the DI. However, in this article we treat such functions as trajectories, and the reachable set of the DI as the solution. This is because the reachable set is a natural generalization of a single solution to a differential equation.

Roughly speaking, the *reachable set* is the *union of the graphs of all trajectories* of the DI. A more exact definition is as follows.

First, recall that the *graph* of a function *f(t)* is the set of all ordered pairs (*t*, *f*(*t*)).

Let *X_o_* be a closed and connected subset of *R^n^, I*⊂*R* denotes an interval *[t_o_,t_1_], x(t)εR^n^* is the model state vector*, F: RxR^n^→P(R^n^)* is a set-valued function, where *P(X)* denotes the power set i.e. set of all subsets of a space *X*.

The reachable or attainable set of (1) is defined as the union of the graphs of all trajectories of (1).

Like the solution to a differential equation with the necessary regularity assumptions, the reachable set exists and is unique. To avoid a conflict of terminology with other sources, we will not use the term solution, discussing rather the reachable sets.

The way we obtain the differential inclusion for a given ODE model with uncertainty is based on the fact that the DIs are closely connected to control systems. Consider an ODE model with uncertain and variable parameter, supposing that we have no, or poor information about the probabilistic properties of the parameter(s), like probability distributions and confidence intervals, but we can assess the intervals where the parameters must belong.

The *control system* associated with differential inclusion (1) is defined as follows.

(2) $$\begin{array}{l} \frac{dx}{dt}=f(x,u,t)\\ x\in R^{n},\;u\in C(x,t),\;C\subset R^{m},\;u\in R^{m},\;t\in I \end{array}$$

where x is the system state, u is the control variable f is a vector-valued function and I⊂R^+^ is a closed time interval. The control variable u represents the uncertain parameter.

When the control u scans its permissible set C (i.e. if it takes all possible values in C), then the (vectorial) value of the function *f* scans a set, which is exactly the set *F* of the inclusion (1). This way, the system (2) defines the differential inclusion. In the sequel, we will use this representation of the Dis. [44].

Note that the reachable set of a DI is a deterministic object. Thus, we replace any stochastic or probabilistic approach with a deterministic one. The two methods can hardly be compared to each other. The advantage of the DI application is that we do not need any probabilistic distributions or properties of the considered uncertain variables, using only the ranges of possible fluctuations. As the result, we obtain the reachable set in the state space, telling us what are possible extreme values and where the model trajectories must belong. It should be emphasized that to assess the reachable set *we do not apply simple perturbances* to the model parameters. To calculate the reachable set we solve the corresponding differential inclusion, which is not an easy task. These issues and the DI modeling are explained with more detail in the following sections.

The *Differential Inclusion Solver* is used to calculate the shape of the reachable set. See Raczynski [20] for detailed description of the solver algorithm. Recall that the main concept of the solver is to scan the boundary, and not the interior of the reachable set. We use the methods of the optimal control theory, namely the Maximum Principle of Pontryagin [21]. However, our problem is not to minimize any particular utility function. What is taken from the Maximum Principle is the fact that the optimal trajectory lies on the boundary of the reachable set. So, we only use the Hamilton-Jacobi equations defined in the Principle, and not the whole optimization procedure.

One could suppose that to assess the shape of the reachable set of a DI we can generate a number of random trajectories of the DI (with simple perturbations) that must belong to its interior. In Raczynski [20] it is pointed out that such simple shooting leads to poor assessments and wrong results. The trajectories shown in the next sections might appear to be random, but remember that these are trajectories that scan the boundary of the reachable set. Each one of these trajectories satisfies Hamilton-Jacobi equations, see Pontryagin [21].

The reachable set should not be confused with the *viability kernel* as defined in see Refs. [2,22,23]. The reachable set and viability kernel are different concepts. Here, we discuss reachable sets and not viability problems.

As stated before, the definition of the reachable set and its properties have been published nearly 80 years ago, and the properties of the trajectories that scan the reachable set boundary are known in the optimal control theory for more than 50 years. What is new in this paper is the application of the DI solver to some economic models with uncertain parameters varying in time. The corresponding software developed by the author permits to show the images of the reachable sets due to the uncertainty, never shown before.

The DIs can be used to assess the influence of uncertainty on the model behavior. Our point is that uncertain parameters need not be random. A random variable has its probability distribution and obeys certain rules of probability theory. In turn, an uncertain parameter may have no distribution and no probabilistic properties. It is just uncertain, and perhaps may change within a given limits. For example, in marketing, the strategy of the competition may be unknown, but we can hardly treat it as random variables. As for the behavior of an uncertain parameter, we can hardly assume that it remains unchanged along the model trajectory. Thus, in the following, we will treat all uncertain parameters as time-dependent variables.

## How the simulations are carried out

The images shown in the following sections show the results of applications of the DI Solver, rather that simple simulations provided by various ODE and System Dynamics packages. To obtain the shape of the reachable set, the differential inclusion of the model is introduced to the Solver program, in the form of the corresponding control system. The control variables of the control system represent uncertain parameters with given restrictions (set C in (1)). Then, the DI Solver generates a number of trajectories that scan the boundary of the reachable set, as explained in sections 2 and 3. The trajectories are stored in a file and then, used to visualize the shape of the reachable set. Each trajectory is the result of solving the ODE system corresponding to each particular model. The model parameters under consideration are not fixed for each trajectory and can fluctuate in time.

Note that the reachable set (RS) is not computed generating all possible trajectories of the DI and looking for the union of the corresponding graphs. Such way of computing the reachable set may appear to be simple and sufficient, but this is not true. Generating millions of (random?) trajectories gives a poor image of the set, and is computationally inviable. The DI Solver computes the boundary of the RS generating trajectories that satisfy Hamilton-Jacobi equations in a similar way as in the optimal control theory. Such trajectories lay on the RS boundary. Having several hundred boundary-scanning trajectories, we can assess the shape of the RS.

We will not discuss here the solver algorithm in more datil, to avoid repetition. The detailed solver algorithm has been already described in Raczynski [20].

## Method steps, Di Solver vs sensibility analysis available in the System Dynamics software

The proposed method consists in the application of differential inclusions to determine reachable sets for the model trajectories in the time-state space. A possible application may be the uncertainty treatment, as described in the following sections. The differential inclusion solver, developed by the author is the main methodological tool. The steps of the application procedure are as follows.

1 Find a dynamical model of the real system to be analyzed, expressed in the form of a set of ordinary differential equations.
2 Identify model parameters that may change along the model trajectory, for example those that are subject to uncertainty. Note that this is not a "Vensim-like" sensitivity analysis because we treat the parameters as functions of time, and not as values fixed along each model trajectory.
3 Apply the differential inclusion solver to get the reachable set where the model trajectories must belong.

Some System Dynamics (SD) packages, like Powersim or Vensim Powersim (see Ref. [24]) offer the risk and sensitivity analysis feature. For some researchers a question may arise, why use the complicated differential equations approach instead of simply running the risk analysis of a SD program. Below we compare the results of the Powersim sensitivity analysis results with the reachable sets generated by the DI Solver.

Consider an example of a simple second order model as follows.

(3) $$\begin{array}{l} \frac{dx_{1}}{dt}=x_{2}\\ \frac{dx_{2}}{dt}=p_{1}-x_{1}-p_{2}x_{2} \end{array}$$

where *x_1_* and *x_2_* are the state variables and *p_1_* and *p_2_* are uncertain parameters. Suppose that the default values are *p_1_* = 1, and *p_2_* = 0.15. The initial conditions for *x_1_* and *x_2_* are both equal to zero, and the simulated time interval is [0, 20]. Simulating this model with Powersim we obtain a simple dumped oscillatory movement that approaches the point (1, 0). Now, suppose that the parameter values are uncertain and obey some restrictions: *p_1_* ε [0.9, 1.1] and *p_2_* ε [0.1, 0.2]. The Powersim risk analysis provides the following results for the state variables, at time = 20: 0.72< *x_1_* <0.98 and 0.16< *x_2_* <0.48.

**Fig. 1:**
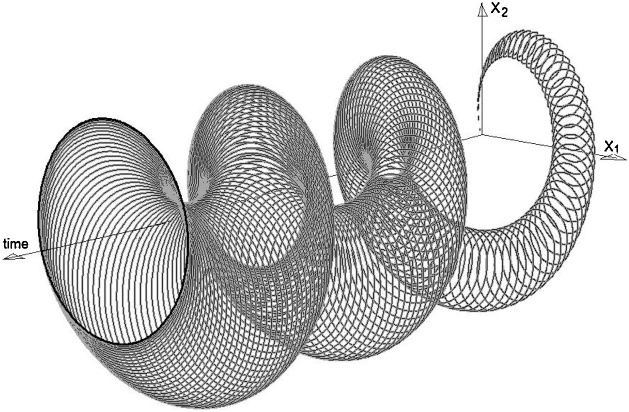
The 3D image of the reachable set for model (3).

**Fig. 2:**
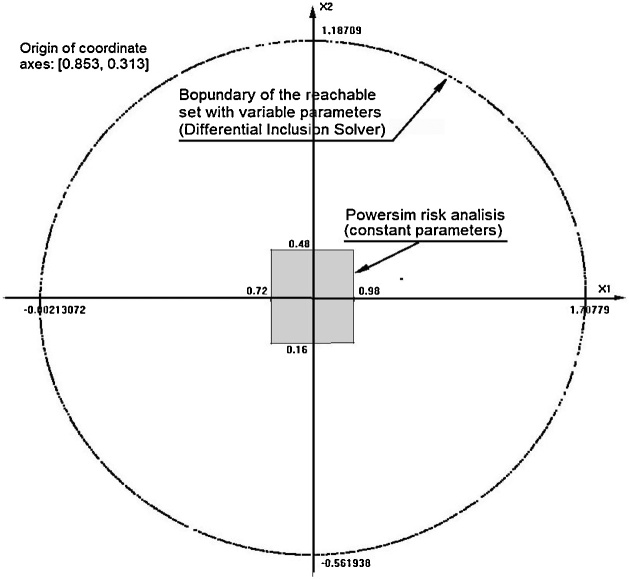
The contour of the reachable set for system (3), calculated by the DI Solver.

Now, let us treat (3) as a differential inclusion. The right-hand side of the inclusion is the set defined by (3), where p_1_ and p_2_ scan their permissible intervals. The resulting reachable set is shown in Fig. 1. This is the 3D image in the space (x_1_, x_2_, time) provided by the DI Solver that shows the consecutive “slices” of the reachable set. Fig. 2 shows the contour of the reachable set for time equal to 20. The small gray rectangle inside the reachable set represents the Powersim results. It can be seen that the DI Solver provides the reachable set several times greater than Powersim risk analysis.

The attainable values for the state variables are −0.002< x_1_ <1.708 and −0.562 < x_2_ <1.187, several times greater than the Powersim assessment. This discrepancy in the risk analysis between DI Solver and Powersim is caused mainly by the fact that in Powersim the parameters remain constant over the trajectory. In the real world, however, this is not necessarily true. For example, in the Lotka-Volterra prey-predator model the parameters may dramatically change due to the environment conditions like the weather, epidemics and human activities. In the economic growth models the changes may be caused by natural disasters or political events.

It might appear that to use parameters fluctuating in time it is sufficient to generate their values randomly in some model time instants, scanning the interior if the reachable set and getting the assessment of its shape. It is no room here to show the corresponding experiments, so only let us mention that such method provides results even worse than the Powersim risk analysis.

The above considerations are not aimed to depreciate the Powersim. Its risk analysis is simple and useful, giving the user a general idea over the model sensitivity. The method is robust and may work with big models of higher order, while the DI solver becomes rather slow when the model size grow. On the other hand, DI approach provides the true attainable sets and should be used always when the parameters fluctuate in time. Another advantage of the DI Solver is that it provides, as a “side product”, the information about model optimal control including the best and worst case.

## Some models of economic growth

The research on economic growth models dated from the 18th century. The early publication “An Inquiry into the Nature and Causes of the Wealth of Nations” of Adam Smith appeared in 1776, now available from other editions [25]. Smith deals with the production and distribution in capitalistic system, being the main theoretic contribution to the pre-Marxist economy. Smith studied the formation of the capital, the productivity and the output from workers. The factor of technical progress and its importance has been addressed later by Ricardo [26].

More mathematical models of economy dynamics appeared in early 20^th^ century. Sir Roy Harrod in 1939 and Evsey Domar in 1946 developed a more detailed model of economic growth (see Ref. [27]). In this model the economy growth is dependent on the level of saving and the capital output ratio (productivity of capital investment). The model explains how growth has occurred and how it may occur again in the future.

An interesting model that shows a constancy in the capital/output ratio, including “capital saving” and “labor saving” factors can be found in Nicolas Kaldor [28]. The Keynesian [29] approach, developed in 1930s is used. The model also takes into account the limited available resources. The model variables are income, capital, profits, wages, investment and savings. As the result, the model provides long run tendencies of the economic growth.

Kaya [30] proposes a 5-stage development model. The process of the economy growth is divided in the stages of (1) traditional society, (2) preconditions for takeoff, (3) economic takeoff, (4) drive to maturity, (5) mass consumption. This is a long-term approach. The traditional society stage is assumed to include the 18th century societies. The takeoff in considered to appear in the 19th century. Drive to maturity takes place in 19th and 20th century. Mass consumption period in US and Canada is located in the 1920th and in other countries after the World War II.

It is out of scope of this article to give more detailed survey, consult, for example, Basu [31]. In this chapter, we will focus on the Solow-Swan model, proposed by Solow [32] and Swan [33], and the Bhattacharya [34] three sector models (see also Diamand and Spencer [35].

Walde [36] addresses the problem of optimal household behavior, including a risk factor, savings and returns. However, the paper is focused on equilibrium rather than the extreme model behavior. Barlevy [46] discusses the design of macroeconomic policies with uncertainty. The aim is to minimize the worse-case. The uncertainty generated by the environment is taken into account. It is pointed out that the worst-case is not the most important issue, compared to the optimization of the macroeconomic policies.

## Solow-Swan and Mankiw-Romer-Weil models: trajectories and reachable sets

The Solow-Swan model is closely connected to the Cobb and Douglas model [37], which states that

(4) $$Y=AL^{\beta }K^{\alpha }$$

Where Y is the total production (value of goods), L is the corresponding labor (person-hour), *K* is the capital used (machinery, buildings, equipment), *A* is the productivity factor, α and β are constants named elasticities. The sum α + β may be equal, less or greater than one. If it is equal to one, the output *Y* depends in linear way on the product *KA*. Is it is greater than one, the output grows faster than *KA*, and if it is less than one, the increased size of *KA* does not provide the proportional production growth.

The Solow-Swan model consists in the following equations:

(5) $$Y={\left(A(t)L(t)\right)}^{\left(1-\alpha \right)}K{\left(t\right)}^{\alpha },\;0<\alpha <1$$

Here, AL represents the effective labor. We introduce the relative values as follows.$y(t)=\frac{Y(t)}{A(t)L(t)},\;k(t)=\frac{K(t)}{A(t)L(t)}$

Capital intensity *k* is the capital stock per unit of effective labor *AL*.

The labor factor and the technology level grow in time, as follows.

(6) $$L(t)=L(0)e^{nt},\;A(t)=A(0)e^{gt}$$

Supposing that only a fraction 0 < c < 1 of the output Y is consumed, we denote s = 1-c, where s represents the investment share.

With this notation, the Solow-Swan model is expressed as follows:

(7) $$\frac{dk(t)}{dt}=sk{\left(t\right)}^{\alpha }-(n+g+\delta )k(t)$$

where δ is the capital depreciation rate. The equilibrium of the model is achieved when

(8) $$\frac{K(t)}{Y(t)}=\frac{s}{n+g+\delta }$$

The Solow-Swan model predicts that the economy will converge to that equilibrium.

There are some modifications of the model. In the extended version there is the concept of human capital *H(t)* that refers to the stock of knowledge, habits, social and personality attributes, creativity. With the human capital added, the model is known as Mankiw-Romer-Weil (MRW) version (9), see Mankiw [38].

(9) $$\left\{\begin{array}{l} \frac{dk}{dt}=s_{k}k^{\alpha }h^{\beta }-(n+g+\delta )k\\ \frac{dh}{dt}=s_{h}k^{\alpha }h^{\beta }-(n+g+\delta )h \end{array}\right)$$

where *h=H/(AL).*

For the estimates of the parameters n,g and δ see Bernanke and Gürkaynak [39].

Further on we will modify this model introducing some additional inertia and calculate the corresponding reachable sets.

Now, we will carry out some experiments with the original model (9), The model parameters was as follows.

*n* = 0.025, *g* = 0.06, δ =0.05. α =0.25, β = 0.2, *s_k_* = 0.5, *s_h_* = 0.45

**Fig. 3:**
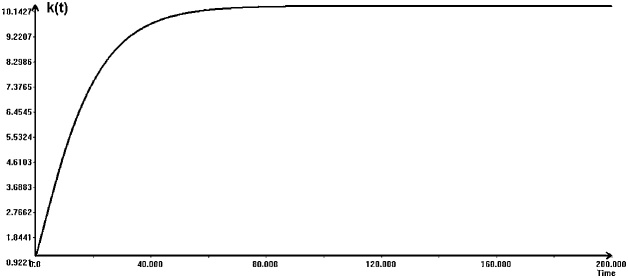
A simple simulation of the Mankiw-Romer-Weil model.

Fig. 3 shows the changes of *k(t)* as the result of a simple simulation. The shape of the curve for *h(t)* is similar, with the steady state (equilibrium) value equal to 9.09. The final time for this simulation was set to 200. It is somewhat long-term simulation, done in order to reach the final equilibrium. In the real world this seems to be too long period. Within 200 years something will probably happen (a war, emerging competitions, etc.) that may influence the economy changes. Further on, while calculating the reachable sets due to uncertainty, the final time is equal to 80.

Now, suppose that some of the model parameters are charged with uncertainty and may change in time. Denote *A = n + g +* δ. We assume that *s_k_, s_h_ A* have uncertain values that may change within and interval of plus and minus 25 percent of their default values.

**Fig. 4:**
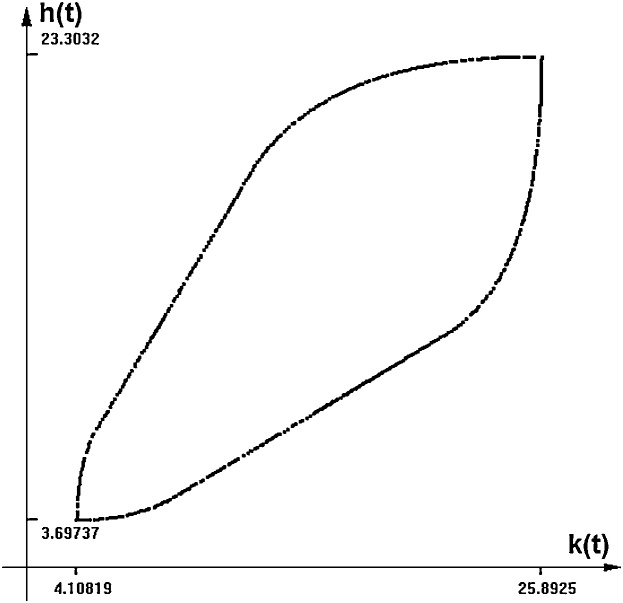
Mankiw-Romer-Weil model. The shape of the reachable set at time = 80.

**Fig. 5:**
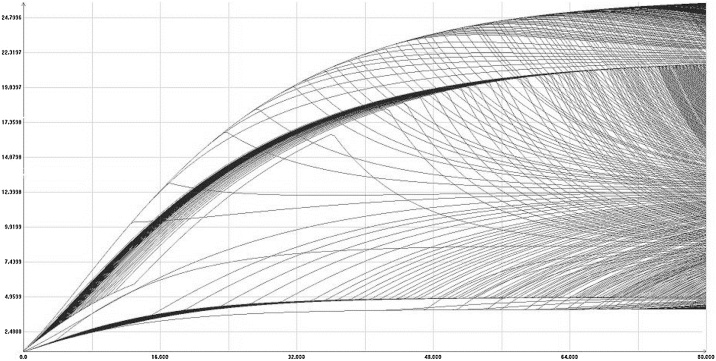
Some boundary scanning trajectories for the MRW model.

In Fig. 4 the time-section of the reachable set at time equal to 80 is shown. Fig. 5 shows some boundary scanning trajectories of the model, projected on the plane *time-k(t)*. Note that these are randomly selected trajectories that lie on the boundary of the reachable set and not random trajectories. It can be seen that the size of the reachable set is greater than the range of the changing parameters.

Note that the range of the extreme values reached by the trajectories is quite big, while the parameters uncertainty is supposed to be of 25% relative value.

## MRW model: additional inertia

Now, let us introduce a small modification of the MRW model. In the MRW model it is supposed that the change in the values of the right-hand side expressions of (9) have immediate effect on the derivatives of *k* and *h*. In the reality, perhaps this influence is not so quick. We will assume some inertia in the model. Namely, let us introduce a first order inertia for both *k* and *h*. Now, the equations become as follows.

(10) $$\left\{\begin{array}{l} \frac{dk}{dt}=p\\ \frac{dh}{dt}=r\\ \frac{dp}{dt}=\left[s_{k}k^{\alpha }h^{\beta }-(n+g+\delta )k-p\right]/T_{1}\\ \frac{dr}{dt}=\left[s_{h}k^{\alpha }h^{\beta }-(n+g+\delta )h-p\right]/T_{2} \end{array}\right)$$

**Fig. 6:**
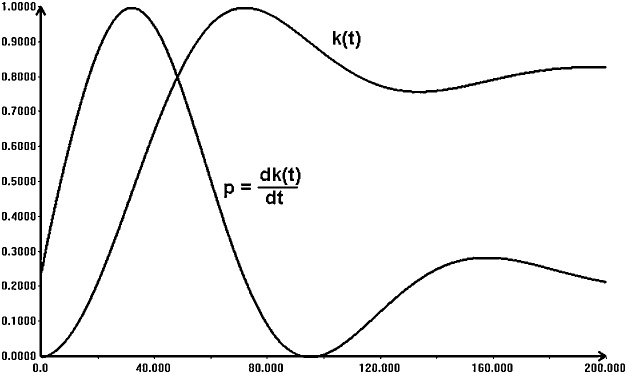
Simple simulation of the MRW model with additional inertia.

The values of the additional variables *p* and *r* follow the changes of the right-hand expressions of (9) with the first order inertia with time constants *T_1_* and *T_2_*, respectively. These, somewhat delayed, values are used to define the derivatives of *k* and *h*. The model is now of the fourth order. Simulating the trajectories without uncertainty, we can see that in this model some oscillations appear. The model is still stable, but it may reflect some tendencies to produce the boom and depression periods. The trajectories of *k(t)* and *p(t)* are shown in Fig. 6. The curves are normalized. The range for *k* is between 1 and 5 and the *p* changes between –0.074 and 0.26.

**Fig. 7:**
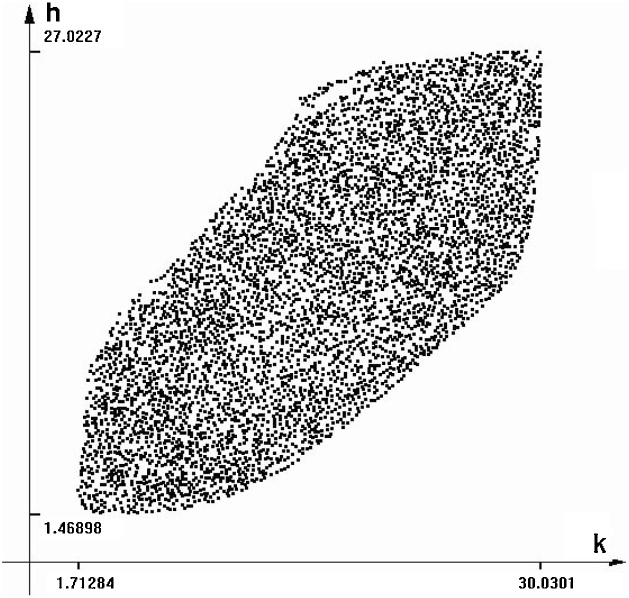
Reachable se for the MRW model with additional inertia. Time = 80.

Differential inclusion solver provides the image of the reachable set for this model, as shown on Fig. 7 (*k-h* plane). As the model is of order four, the solver does not produce a simple contour. What we see is a projection of a 4-dimensional cloud of points on the plane *k-h*. Here, as in the previous experiments, we assume that *s_k_, s_h_* and *A = n + g +* δ have uncertain values that may change within and interval of plus and minus 25 percent of their default values.

**Fig. 8:**
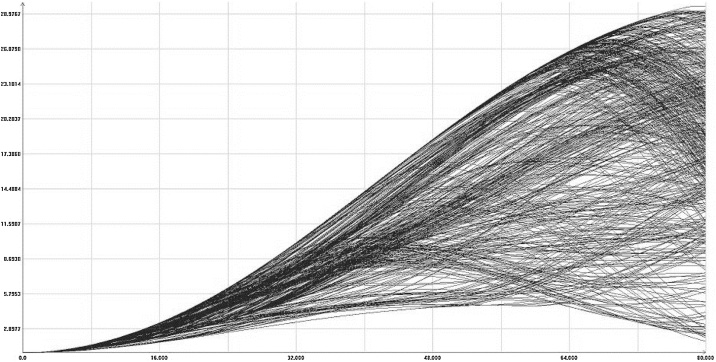
Some boundary scanning trajectories of the MRW model with inertia. Vertical axis is *k(t)*.

It is difficult to visualize a 3-dimensional object like a point cloud on a 2-dimensional figure. The differential inclusion solver provides an option that shows the cloud rotating around one of the axes. This produces a nice view of the rotating reachable set, and permits to observe its boundary. In Fig. 8, we can see some boundary scanning trajectories.

Differential inclusion solver calculates a set of trajectories that scan the boundary of the reachable set. As mentioned before, each of these trajectories is a solution to a problem of optimization with some optimality criterion. Though the main feature of the solver is not optimization, optimization is a “side product” of the program. For each trajectory calculated during the calculations, its parameters are stored. Thus, we can see the control functions (variable parameters history) for any point selected from the reachable set. The user simply selects a point from the time section of the reachable set using the mouse, and the program shows the corresponding control function and the plot of the trajectory over the given time interval.

## The Bhattacharya three sector model: simulation results

In this model three sectors of economy are considered: the rural sector, urban formal sector and urban informal sector. The main purpose of the model, described in the article of Bhattacharya [34], was to assess the value of the Gini (1927) inequality coefficient. The state variables of the model are the number of rural hirers, migration from rural to urban area and the total labour in the urban area. The model is quite complicated with more than 30 parameters and other variables. The dynamic version of the model is given. Here, we use the dynamic version of the Bhattacharya model and treat it with the differential inclusions, in order to see the influence of the uncertainty in models parameters.

The paper of Bhattacharya [34] addresses income distribution in the context of development of an economy with an informal sector. He also considers the migration of both low and high skilled workers from the rural to the urban area. The worker’s migration from rural to urban areas is simulated, see Refs. [[40], [41], [42]].

In Bhattacharya [34] the simulations are focused mainly on the problem of income distribution, given by the Gini coefficient. Recall that the Gini coefficient is the most commonly used measure of inequality. It was developed by the Italian statistician and sociologist Corrado Gini (see Ref. [43]). A Gini coefficient of zero expresses perfect equality. If the Gini coefficient is equal to one (or 100%), then we have the maximal inequality among values (e.g., for a large number of people, where only one person has all the income or consumption, and all others have none). However, in our simulations we use the dynamic version of the model, looking for the behavior of the variables that describe the worker’s migration and employment in the two economy sectors.

The model consists of three ordinary differential equations that describe the changes in the number of rural hirers (h), the number of manual labourers (m) and the total labour in the urban area (L). The hirers are those who own land and the manual labourers do not.

The meaning of variables and parameters is given below.

*h* – number of rural hirers

*m* – number of manual laborers in rural sector

*L* – total labor in the urban area

v* - minimal wage in formal sector

*w* – the wage in the rural sector

*v* – the wage in informal sector

*t* - time

*F_H_, F_m_* functions defined in (12), below

*f* – number of workers employed in the formal sector (see 12)

*A*_h_, *A*_m_, *β*_h_, *β*_m_, *β*_L_, *η*_L_, *η*_m_, *η*_h_, *η*_t_, *ξ*L, *ξ*_f_, *ξ*_t_,g,

*γ*_m_, *γ*_L_, *γ*_t,_ *D*_m_, *D*_h_, *D*_L_, *D*_f_, *D*_t_, *α*_0_, v_0_, w_0_, f_0_, *Π*_ho_ - constants

The model equations due to Bhattacharya are as follows.

(11) $$\left\{\begin{array}{l} \frac{dh}{dt}=\beta_{h}h-g\left(\frac{F_{H}h}{H}\right)\frac{df}{dt}\\ \frac{dm}{dt}=\beta_{m}m-F_{m}m\\ \frac{dL}{dt}=\beta_{L}L+F_{m}m+g\left(\frac{F_{H}h}{H}\right)\frac{df}{dt} \end{array}\right)$$

(12) $$\left\{\begin{array}{l} F_{H}=A_{h}(v^{*}-\Pi_{ho}-\eta_{L}L-\eta_{m}m-\eta_{h}h-\eta_{t}t\\ H=\alpha_{0}L+F_{H}h\\ F_{m}=A_{m}(v-w)\\ v=v_{0}+\zeta_{L}L+\zeta_{f}f+\zeta_{t}t\\ w=w_{0}+D_{m}m+D_{h}h+D_{L}L+D_{f}f+D_{t}t\\ f=\frac{\gamma_{L}L}{v^{*}}+\gamma_{t}t+f_{0} \end{array}\right)$$

Note that in the Eq. (11) appears a term *df/dt.* To avoid numerical differentiation we obtain, as a consequence of the last equation of (12), the following (supposing that γ_L_, γ_t_ and v* are constants).

(13) $$\frac{df}{dt}=\frac{\gamma_{L}}{v^{*}}\;\frac{dL}{dt}+\gamma_{t}$$

Substituting this to the third equation of (11) and resolving with respect to *dL/dt* we gets

(14) $$\frac{dL}{dt}=\frac{\beta_{L}L+F_{m}m+\gamma_{t}gF_{H}h/H}{1-\gamma_{L}gF_{H}h/(Hv^{*})}$$

The last equation replaces the third equation of (11). The term *df/dt* in the first one of (11) is replaced by (13), using the value of *dL/dt*. The equations become as follows (the order of calculations changed in order to define *dL/dt* before using it).

(15) $$\left\{\begin{array}{l} \frac{dL}{dt}=\frac{\beta_{L}L+F_{m}m+\gamma_{t}gF_{H}h/H}{1-\gamma_{L}gF_{H}h/(Hv^{*})}\\ \frac{dh}{dt}=\beta_{h}h-g\frac{F_{H}h}{H}\left(\frac{\gamma_{L}}{v^{*}}\;\frac{dL}{dt}+\gamma_{t}\right)\\ \frac{dm}{dt}=\beta_{m}m-F_{m}m \end{array}\right)$$

**Fig. 9:**
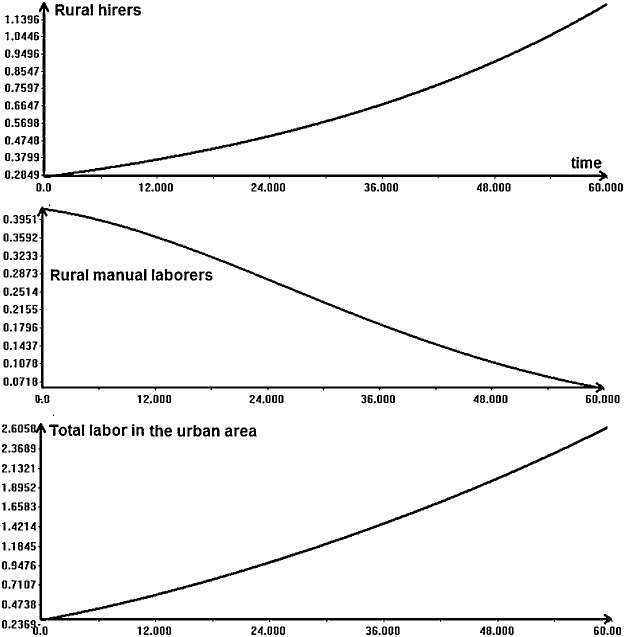
Simple simulation of the model (18).

First, we run a simple simulation. Fig. 9 shows the plots of the variables *h, m* and *L* in function of time. We will not discuss here the numeric values of the variables and parameters. The parameters and initial conditions for the simulation are taken from the article of Bhattacharya [34]. Only observe that the number of manual workers in the rural area decreases, while the labor in the urban area grows.

Now, we apply the DI solver to see the reachable set, supposing that there is some uncertainty in the model. Note that the model has more than 30 parameters, each of them with a possible uncertainty. To explore all the possible uncertainties we would carryout tens of experiments resulting in up to a hundred images. Let us show only some of the experiments that may give a general view on the model behavior.

**Fig. 10:**
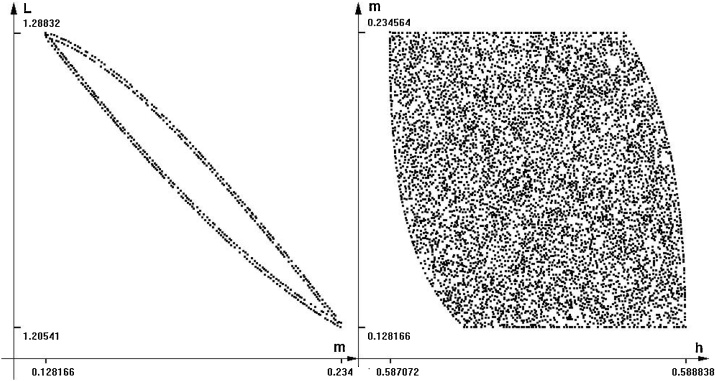
Projections of the reachable set for model (15). Time = 30.

Suppose that the values of variables *w, v* and *H* of the model are not exactly defined and may fluctuate (changing in model time), in the range of plus minus 10 percent each one, along the model trajectory. Fig. 10 presents the images of the reachable set projections on planes *h-m* and *m-L*.

The images of the reachable set give us an assessment of the model sensibility to the dynamic parameter changes. Remember that the selected parameters are not constant along the trajectories.

In the similar way as has been done for the MWR model, we will introduce an additional inertia to the model. Namely, suppose that the migration *m* is charged with the first order inertia with time constant *T*. The inertia means that the changes of the term *F_m_m* (see Eq. (15)) do not affect the derivative of *m* immediately. This may be explained by the delay in the worker’s decision to migrate to the urban area. Now, we have four model equations, as follows.

(16) $$\left\{\begin{array}{l} \frac{dL}{dt}=\frac{\beta_{L}L+q+\gamma_{t}gF_{H}h/H}{1-\gamma_{L}gF_{H}h/(Hv^{*})}\\ \frac{dh}{dt}=\beta_{h}h-g\frac{F_{H}h}{H}\left(\frac{\gamma_{L}}{v^{*}}\;\frac{dL}{dt}+\gamma_{t}\right)\\ \frac{dm}{dt}=\beta_{m}m-q\\ \frac{dq}{dt}=\frac{F_{m}m-q}{T} \end{array}\right)$$

**Fig. 11:**
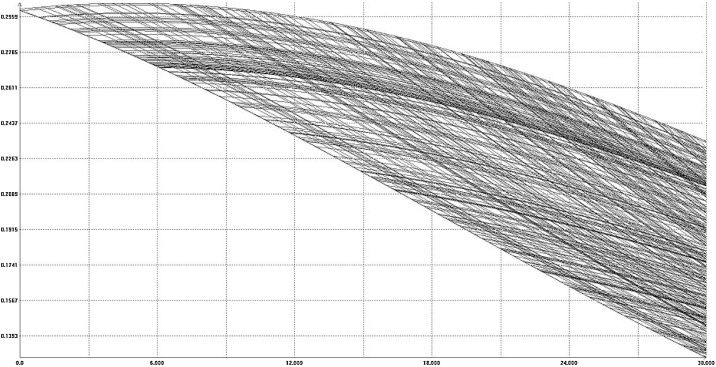
Some boundary scanning trajectories *m(t)* for model (18).

**Fig. 12:**
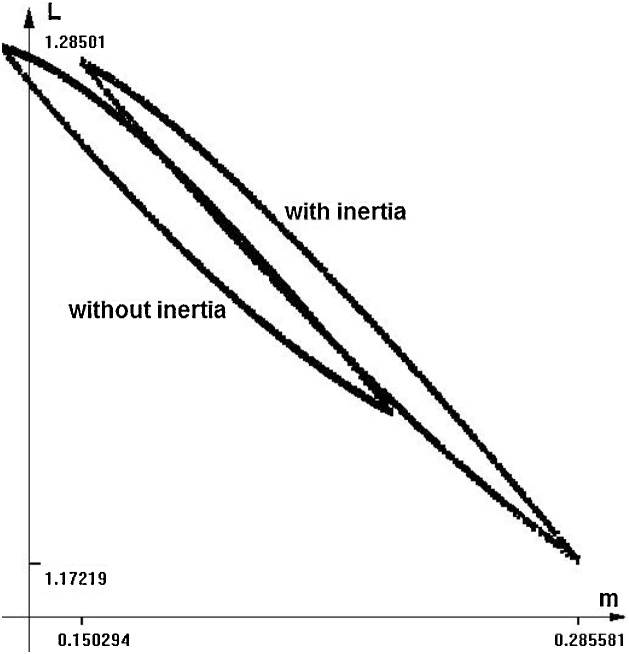
Comparison of the shape of the reachable set between case without and with inertia. Time = 30.

The last equation of (16) represents the inertia. The auxiliary variable *q,* with initial condition zero, is “delayed” with respect to the expression *F_m_m* with time-constant *T*. As for the time-constant *T*, a reasonable value could be equal to one (“will migrate next year”, approximately). However, to show the influence of the inertia, we put T = 5, somewhat exaggerated value. The image of the reachable set in this case is similar to that of Fig. 10, but the values change significantly. In Fig. 11 we can see a projection of some boundary scanning trajectories, without inertia. Fig. 12 shows the comparison of the case without and with inertia, in the same scale.

It can be seen that the maximal value of *m* at the final time grows because some workers delay their migration to the urban area. The shape of the reachable set also changes.

Such changes caused by the inertia imposed over other terms of the model equations can be easily simulated and shown graphically. Perhaps such modifications, with somewhat more complicated dynamics, could give us a wider view on the dynamics of the whole system.

**Fig. 13:**
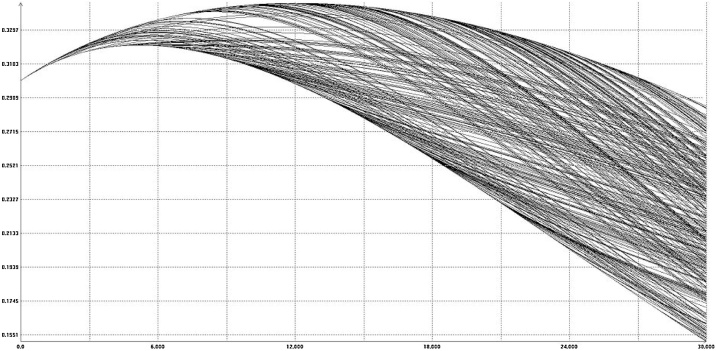
Some boundary scanning trajectories. Additional inertia added.

Fig. 13 shows some boundary scanning trajectories for the case with additional inertia, projected on the plane *t-m*.

## Conclusions

Differential inclusions may be useful in the treatment of uncertainty in system dynamics models. The main difference between the probabilistic approach and that of the differential inclusions is that the concept of the reachable set and the simulation results are deterministic. The differential inclusion solver provides useful information of the model behavior, different from that obtained from the classical differential equation simulations. It can be seen that the reachable sets, due to the uncertain parameters, maybe several times greater than the range of parameter fluctuations. In addition, we can obtain the optimal trajectories, as a “side product” of the solver. It should be emphasized that to assess the reachable set shape *we do not apply simple perturbances* to the model parameters. To calculate such set we solve the corresponding differential inclusion, which is not an easy task. The differential inclusion solver produces trajectories that scan the boundary of the reachable set and not its interior. The information about the shape of the system reachable set may be useful in the evaluation of model sensibility and the influence of uncertainty. Perhaps, in the future, the DI option of system dynamics models might be included in the standard SD modeling software.
